# Supplementary material for: Identification and characterisation of a phospholipid scramblase in the malaria parasite Plasmodium falciparum
Source: Mol Biochem Parasitol. 2021 May;243:111374. doi: 10.1016/j.molbiopara.2021.111374 (PMC8202325; doi:10.1016/j.molbiopara.2021.111374)
Supplement: Supplementary file 1 [file mmc1.pdf]

Figure S1

```
PfPLSCR       --------------------------------MEEKNIHMQPNINYSYRNPNMYNMNY--  26
VbraPLSCR2    --------------------------MKGEKGAEPQQMVMQ---------PQAYGQPQVM  25
VbraPLSCR1    MPQQPTPVAPKAVQPSQSPNAVPPQQIPNHAGMHPTPIPNHAGFASAPPQHQMVGQ--TA  58
CvelPLSCR     ------------------------------------------------------------  0

PfPLSCR       HNPIVPPPQQQMQLFVNDWKSILSPMQTCKIKQQFDDREFVADYFMGFKLDFNNKYLILD  86
VbraPLSCR2    QPVAQMQMQPGMMVINTDPWAILSGMTGAEIKERVRM----AEAILGW--EQNNIYDIKD  79
VbraPLSCR1    TAADAPTPNPAMALGSPDPFIALSGMNKVQVSEKVRA----LEAVIGW--EQNNVFRIKN  112
CvelPLSCR     ------------MQFTMDPFQMLASQQKVYVKEKIKI----L-EFIGI--DQANKYAVKD  41
                               *    *:      :.::.         .:*   :  * : : :

PfPLSCR       ASTELMKFTACEKSEFCYR----NCLPKICIPMNMKILS--YGKEISKPDILMEKDCTCT  140
VbraPLSCR2    QNG-NPAFIAKEDTDTCTRLIGMCCPVQDCRMFKLDISVPPMPGASPPPFLHLDRPWTCT  138
VbraPLSCR1    EFG-ADVMVAKEETEWYKR----NCCLGDCRPWDVDVALCPVAGAPPAPFIHLQRKWSLT  167
CvelPLSCR     VNG-NEMFIAKETTGFCAR----CCLPTTCRPFNLEMF-IKVPGQSPQPFIKADKECQMT  95
                     : * * :    *     *    *   .:.:           * :  ::    *

PfPLSCR       FLCFNRPTIKMYDFSNNNNKELIGTIKTPYRCCSYNFNLFDPSNNKIMYMDDTCCQMSIL  200
VbraPLSCR2    CYCFNRPKVTVTDLTA--GGEVIGTMRDPWHCCNYNMRMADPEGKDVLNVEASCCQCGFW  196
VbraPLSCR1    CCCIGRPKVTVTDVTT--N-QKLGSVSDPWHCCDVTFVIRDHQGRKIMRVRGGGCQCGWC  224
CvelPLSCR     CCCLNRPKMQVMNNHT--G-QQIGILKDDFDCCNHIFSINDNQENPTLRIKGNCMQCGIC  152
                *:.**.: : :     . : :* :   : **.  : : * . .  : :     * .

PfPLSCR       CPCPWGPFKYSNFFLRDANSKEKIAHLRKEVP--FLKFVKRDIDNYTLDFEQVQNPEWKM  258
VbraPLSCR2    CPLPCGPCKEVQFEITDVESKQKVGHFKRIVPCNLLKFLFTDVDNYQVEFGGITDPKWKS  256
VbraPLSCR1    CHCPCGPCRKIKFPVFDAKTGREIASIRHEWGG-LFKSMCTDADDYWIDFGEVQDPRWKS  283
CvelPLSCR     CKCPCGPCKYVKFDITDMKSQQAVGQIVKENTC-LGTIADDMNDKYWVDFGAVSEPQWKA  211
              *  * ** :  :* : * :: . :. : :     : .      *.* ::*  : :*.**

PfPLSCR       MLLAFALFLDYMYYDRK-----------                                  275
VbraPLSCR2    LLLAAAVFADFRYFSTNKNENSALGTIS                                  284
VbraPLSCR1    LLIATALFLDFRYFSRGADGLAA-----                                  306
CvelPLSCR     MLVTAAIFIDFRMFQGGKKDENSTG---                                  236
              :*:: *:* *:  :.
```

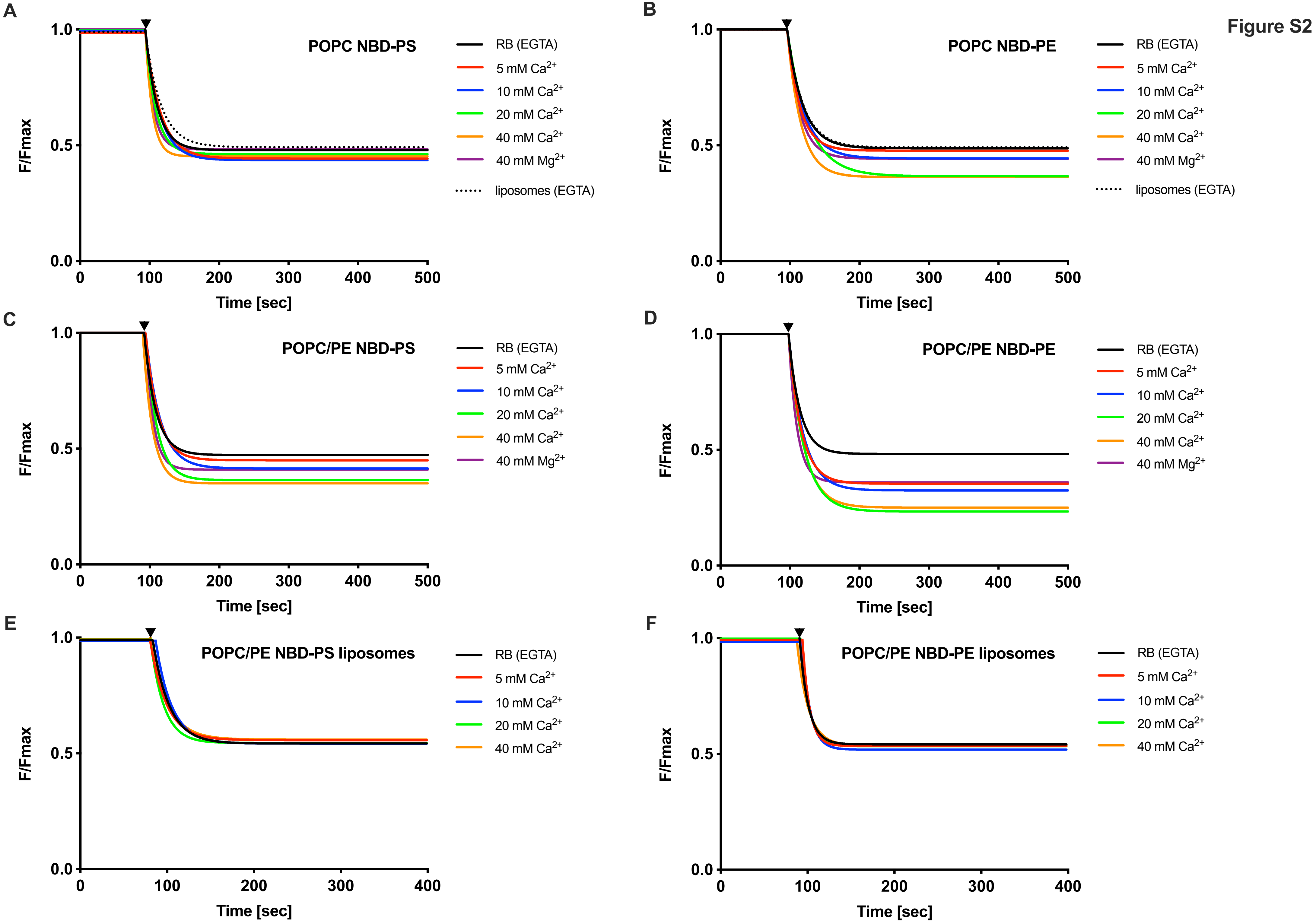
Figure S2
A
POPC NBD-PS
B
POPC NBD-PE
C
POPC/PE NBD-PS
D
POPC/PE NBD-PE
E
POPC/PE NBD-PS liposomes
F
POPC/PE NBD-PE liposomes
RB (EGTA)
5 mM Ca2+
10 mM Ca2+
20 mM Ca2+
40 mM Ca2+
40 mM Mg2+
liposomes (EGTA)
F/Fmax
Time [sec]

Figure S3

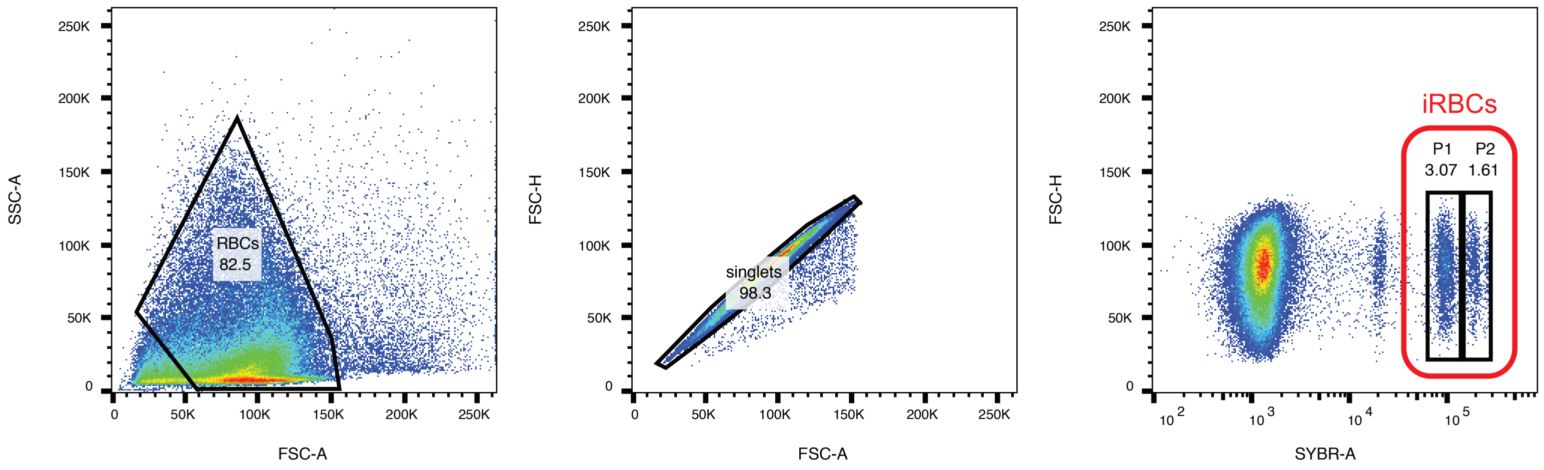

SSC-A
250K
200K
150K
100K
50K
0
RBCs
82.5
0
50K
100K
150K
200K
250K
FSC-A
FSC-H
250K
200K
150K
100K
50K
0
singlets
98.3
0
50K
100K
150K
200K
250K
FSC-A
FSC-H
250K
200K
150K
100K
50K
0
iRBCs
P1
3.07
P2
1.61
10^2
10^3
10^4
10^5
SYBR-A

Table S1. All primers used in the study.

1022700 cKO & KO P1	GAACATAAATATATTTAAAATGTTATATATACATAGATAG
1022700 cKO & KO P2	GAGAAGGTTATTGCATCCTTCAATAAAATGGGAAAATTG
NeoR P3	GGCCAGCCACGATAGCCGCGCTGCCTCG
GFP P4	CAAGTGTTGGCCATGGAACTGGTAGTTTTCCAGTAGTGC
M13R P5	CAGGAAACAGCTATGACCATG
1022700 5F (XhoI)	CGCctcgagGGTATTTCCCAGGTGCTCGT
1022700 5R (HindIII)	CGCaagcttCATATGTATATTTTTCTCTTCC
1022700 3F (NotI)	CGCgcggccgcGCTCACCTACGTAAAGAAGT
1022700 3R (SacI)	CGCgagctcGGTTTAAACAAAATATGCAG
guide952530 S	TATTCGATAACTGTAATTTATATT
guide952530 AS	AAACAATATAAATTACAGTTATCG
guide952497 S	TATTGTGGGGGCACTATAGGGTTA
guide952497 AS	AAACTAACCCTATAGTGCCCCCAC
guide951969 S	TATTATCTTATGCCCTTGTCCATG
guide951969 AS	AAACCATGGACAAGGGCATAAGAT
1022700 KO P6	GACAATAAATATATTTTCTTTAACC
1022700 KO P7	GTGTGACATTTATGTGTAAATG
1022700 KO P8	GAGAATAACGACCGATATTTTATC
1022700 KO P9	GAACAAGAACAAAGTTCTAATTAG
