# Supplementary material for: Identification and characterisation of a phospholipid scramblase in the malaria parasite Plasmodium falciparum
Source: Mol Biochem Parasitol. 2021 May;243:111374. doi: 10.1016/j.molbiopara.2021.111374 (PMC8202325; doi:10.1016/j.molbiopara.2021.111374)
Supplement: Supplementary file 2 [file mmc2.docx]

1. **Supplementary Information**

**Figure S1. *Pf*PLSCR is conserved in unicellular algae.**

Alignment of *Pf*PLSCR with the orthologous proteins from the closely related *Chromera velia* and *Vitrella brassicaformis* algae. Similar residues are marked by colons (:) and identical residues are indicated by stars (*). Protein sequences were aligned with Clustal Omega [[77](#_ENREF_77)]. Predicted bipartite nuclear leader sequences are highlighted in yellow, putative Ca^2+^ binding regions in green and the C-terminal transmembrane helices are underlined. The cut-off score was set to 4.0 for the prediction of palmitoylation sites [[49](#_ENREF_49)] highlighted in orange.

**Figure S2. PL scrambling by recombinant *Pf*PLSCR.**

Representative traces are shown for each PL composition. The translocation of **A & C)** NBD-PS and **B & D)** NBD-PE in *Pf*PLSCR containing proteoliposomes was recorded in the presence and absence of Ca^2+^ and Mg^2+^ ions, respectively. Protein-free liposomes are denoted by dotted lines. **E & F)** Traces of empty POPC:POPE (70:30) liposomes in the presence of different Ca^2+^ concentrations. The decay of fluorescence is plotted as F/F_max_ with F= fluorescence at Time [sec] and F_max_= fluorescence prior to addition of 30 mM sodium dithionite (indicated by arrow heads at 100 sec).

**Figure S3. Gating strategy used to analyse flow cytometry data.**

The populations P1 and P2 denote single (P1) and multiply ring-stage infected red blood cells (P2). Rings from the P2 population were counted as one.
